# Elevated pulse pressure and cardiovascular risk associated in Spanish population attended in primary care: IBERICAN study

**DOI:** 10.3389/fcvm.2023.1090458

**Published:** 2023-05-09

**Authors:** Ana Moyá-Amengual, Antonio Ruiz-García, Vicente Pallarés-Carratalá, Adalberto Serrano-Cumplido, Miguel Ángel Prieto-Díaz, Antonio Segura-Fragoso, Sergio Cinza-Sanjurjo, the researchers of the IBERICAN study, Alfonso Barquilla García

**Affiliations:** ^1^ Occupational and Physical Education and Sports Physician, Santa Catalina Health Centre, Palma, Spain; ^2^ Lipids and Cardiovascular Prevention Unit, Pinto University Health Centre, Madrid, Spain; ^3^ Health Surveillance Unit, Mutual Insurance Union, Castellón, Spain; ^4^ Department of Medicine, Jaume I University, Castellon, Spain; ^5^ Working Group of Hypertension and Cardiovascular Disease, Semergen, Madrid, Spain; ^6^ Vallobín Health Centre, Oviedo, Spain; ^7^ Castilla-La Mancha University, Toledo, Spain; ^8^ Milladoiro Health Center, Santiago de Compostela, Spain

**Keywords:** hypertension, pulse pressure, cardiovascular risk factors, subclinical target organ damage, hypertensive cardiovascular disease, cardiovascular disease

## Abstract

**Introduction:**

Elevated pulse pressure (ePP) is an independent marker of cardiovascular risk (CVR) in people older than 60, and a functional marker of subclinical target organ damage (sTOD) which can predict cardiovascular events in patients with hypertension (HTN), regardless of sTOD.

**Objective:**

To evaluate the prevalence of ePP in adult population seen in primary care and its association with other vascular risk factors, sTOD and with cardiovascular disease (CVD).

**Materials and methods:**

Observational multicentre study conducted in Spain (8,066 patients, 54.5% women) from the prospective cohort study IBERICAN recruited in Primary Care. Pulse pressure (PP) was defined as the difference between the systolic blood pressure (SBP) and the diastolic blood pressure (DBP) ≥60 mmHg. Adjusted (for age and sex) ePP prevalence were determined. Bivariate and multivariate analyses of the possible variables associated with ePP were carried out.

**Results:**

The mean of PP was 52.35 mmHg, and was significantly higher (*p* < 0.001) in patients with HTN (56.58 vs. 48.45 mmHg) The prevalence of ePP adjusted for age and sex was 23.54% (25.40% men vs. 21.75% women; *p* < 0.0001). The ePP prevalence rates increased linearly with age (*R*^2^ = 0.979) and were significantly more frequent in population aged ≥65 than in population aged <65 (45.47% vs. 20.98%; *p* < 0.001). HTN, left ventricular hypertrophy, low estimated glomerular filtration rate, alcohol consumption, abdominal obesity, and CVD were independently associated with ePP. 66.27% of patients with ePP had a high or very high CVR, as compared with 36.57% of patients without ePP (OR: 3.41 [95% CI 3.08–3.77]).

**Conclusions:**

The ePP was present in a quarter of our sample, and it was increased with the age. Also, the ePP was more frequent in men, patients with HTN, other TOD (as left ventricular hypertrophy or low estimated glomerular filtration rate) and CVD; because of this, the ePP was associated a higher cardiovascular risk. In our opinion, the ePP is an importer risk marker and its early identification lets to improve better diagnostic and therapeutic management.

## Introduction

Hypertension (HTN) is an important cardiovascular risk factor (CVRF), both at individual and population levels (1, 2). Its control is important because, after nutritional alterations, HTN ranks second in terms of factors responsible for both worldwide mortality and years of life lost and disability-adjusted life years (3). Its association with other factors multiplies the cardiovascular risk (CVR) (4), which justifies the multifactorial approach to these patients.

The pulse pressure (PP) is an index of the distensibility of the great arteries, and therefore it is a functional marker of subclinical target organ damage (sTOD), it predicts cardiovascular events in patients with HTN (5, 6), and it is an independent marker of CVR in population aged >60 (7).

From a pathophysiological point of view, the early phases of HTN are characterised by changes in the blood circulation of the small blood vessels caused by the systemic vascular resistance. The stiffness of the great vessels increases and they lose elasticity as they age, so a greater pressure is needed which causes left ventricular hypertrophy (LVH). In adults aged <55, the increased vascular resistance results in elevated systolic blood pressure (SBP) and diastolic blood pressure (DBP), and both are predictors of cardiovascular disease (CVD). On the other hand, in people aged >55, the DBP tends to increase until it reaches its peak at the age of 55–60, and then it decreases, in such a way that whereas the DBP decreases, the SBP continues to increase. This would explain the fact that an elevated DBP on its own is less useful as a CVR predictor in older patients, while the prediction of the SBP for CVD is maintained (8).

Kodama et al.'s meta-analysis (9) showed that, in patients with DM, for each 10 mmHg increase, the PP had a higher relative risk of CVD than the SBP, DBP and mean BP. The 33-year follow-up study of the Chicago Heart Association Detection Project (10) showed the predictive usefulness of the PP when it is associated with cerebrovascular disease, coronary heart disease (CHD) and heart failure (HF). The elevation of the PP, caused progressively by ageing, was associated with LVH, albuminuria, carotid intima-media thickness (11) and CVD (12). All this caused that, with age, the elevated pulse pressure (ePP) was more closely correlated with SBP (5, 7, 13).

Like other biological variables, PP is a continuous variable that can increase the absolute risk of cardiovascular events in older subjects (≥50) despite the observed decrease in the relative risk (14). It can also be an independent predictor of mortality from any cause and of coronary origin, especially when the PP reaches values ≥65 mmHg (15), being more remarkable in patients with HTN with high levels of PP (13).

It should be noted that the population with HTN and very high CVR [with diabetes mellitus (DM) and/or previous CVD] has higher values of PP than the rest of patients without DM or CVD (16, 17). Moreover, the hypertensive patients with a PP ≥65 mmHg present LVH or echocardiographic diastolic dysfunction more frequently than those with PP <65 mmHg (18). Finally, a significant proportion of treated hypertensive patients have increased arterial stiffness, a finding that can partly explain the remarkable residual risk of CVD associated even with a well-controlled HTN (19).

It is very important to consider a comprehensive approach to CVR in all patients in primary prevention, before the development of CVD, and which includes the determination of the PP together with the rest of main CVRF. In order to increase knowledge in this regard, the objective of this study is to evaluate, in the context of the IBERICAN study, the presence of ePP in population seen in Primary Care, and its association with other CVRF, sTOD and CVD.

## Methods

An observational, cross-sectional analysis was carried out from the inclusion visit of the IBERICAN cohort, which is a multicentre study conducted in Primary Care centres of the Spanish National Health System and whose methodology has been previously published (20). Using consecutive non-probability sampling, 8,066 subjects aged 18–85 were recruited in Primary Care, who consulted their family physician for whatever reason. Blood Pressure was measured with calibrated devices commonly available in clinical practice. The ePP was defined as the difference between SBP and DBP ≥60 mmHg. The rest of variables considered in this study can be found in the additional material.

### Statistical analysis

The statistical analysis was performed with the program SPSS® (IBM Corp. Released 2015. IBM SPSS Statistics for Windows, Version 23.0. Armonk, NY: IBM Corp.). The qualitative variables were analysed with frequency distribution, percentages, chi squared test, and odds ratios (OR). The continuous variables were evaluated with the determination of the arithmetic means with standard deviation (±SD), median and interquartile range (IQR) of the variables age and PP, *t*-Student test or analysis of variance. The association between variables was estimated with a 95% confidence interval (95% CI) and level of significance *p* < 0.05. The crude prevalences and prevalences adjusted for age and sex were determined through direct method, using standardized 10-year age groups according to the information on the Spanish population aged 18–85 provided by the National Institute of Statistics on July 1st 2021 (21).

To assess the individual effect of comorbidities and CVRF on the dependent variable ePP, a binary unconditional multivariate logistic regression analysis was performed using the backward stepwise method, initially introducing into the model all the variables which showed association in the univariate analysis up to a value of *p* < 0.10, except the variables age, sex and CVR categories which were analyzed individually. The distribution of the specific rates of ePP prevalence by 10-year age groups was analysed using linear regression. Collinearity was previously examined through Spearman's rank correlation coefficient. The model included the variables that showed correlation coefficients higher than 0.5. Then, the variable that contributed least to the adjustment of the analysis was eliminated in each step. All the tests were considered to be statistically significant if the two-tailed *p* value was lower than 0.05. A literature search was carried out on PubMed, Medline, Embase, Google Scholar and Web of Science to compare this study with other similar studies published since 1997.

## Results

The IBERICAN cohort included 8,066 subjects aged between 18 and 85 (54.5% women), with a mean (±SD) age of 58.41 (±14.83) and a median (IQR) of 59.77 (48.97–69.91) years. The mean (±SD) and the median (IQR) of the PP of the study population were 52.35 (±12.95) mmHg and 50 (43–60) mmHg respectively, where the mean PP was significantly higher (*p* < 0.001) in men [53.65 (±12.50) mmHg] than in women [51.26 (13.21) mmHg]. In patients with HTN, the mean (±SD) and the median (IQR) of the PP were 56.58 (±13.22) mmHg and 55.0 (49.0–64.0) mmHg respectively, where the mean PPs were practically the same (*p* = 0.981) in men [56.58 (±13.17) mmHg] and in women [56.57 (±13.29) mmHg]. In patients without HTN, the mean (±SD) and median (IQR) of the PP were 48.45 (±11.37) mmHg and 50.0 (40.0–55.0) mmHg respectively, where the mean PP in men [50.31 (±10.77) mmHg] was significantly higher (*p* < 0.001) than in women [47.16 (±11.60) mmHg].

The crude prevalence of ePP was 30.03% (95% CI 29.03–31.04), being significantly different (*p* < 0.001) in men [32.55% (95% CI 31.04–34.10)] and in women (27.92% [95% CI 26.60–29.27]). The prevalence of ePP adjusted for age and sex was 23.54% (25.40% in men; 21.75% in women).

**Figure 1:**
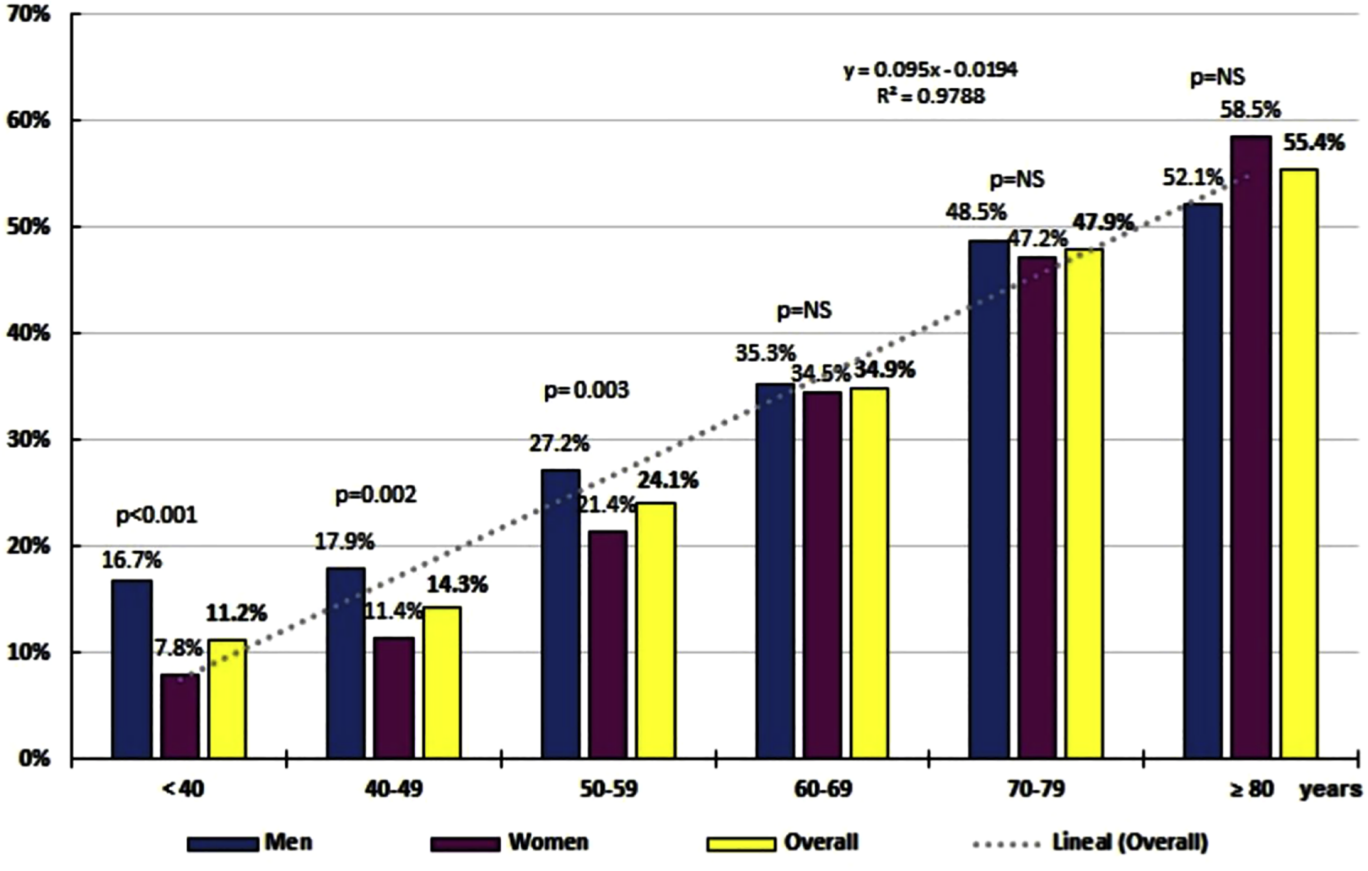
Linear correlation between prevalence of elevated pulse pressure (ePP ≥60 mmHg) and age of the IBERICAN cohort.

The distribution of specific rates of ePP prevalence by 10-year age groups increased with age in a clear way (*R*^2 ^= 0.979) according to the function *y* = 0.095x–0.0194, being significantly higher in men up to the age group 50–59, and without significant differences in the oldest age groups (Figure 1). The OR of the prevalence of ePP between the populations aged ≥65 and <65 was 2.57 (95% CI 2.23–2.96). The prevalence of ePP in patients aged ≥65 was 45.47% (95% CI 43.67–47.26), which was similar (*p* = 0.983) in men (45.49% [95% CI 42.88–48.09]) and women (45.45% [95% CI 42.99–47.92]). The prevalence of ePP in population aged <65 was 20.98% (95% CI 19.86–22.12), which was significantly different (*p* < 0.001) in men (24.50% [95% CI 22.73–26.28]) and in women (18.16% [95% CI 16.74–19.58]).

The clinical characteristics of the patients with and without ePP are shown in Table 1. All the variables were significantly higher in the population with ePP, except height, total cholesterol and non-HDL cholesterol (with non-significant differences), and the estimated glomerular filtration rate (eGFR) and HDL cholesterol (significantly higher in patients without ePP).

**Table 1:** Clinical characteristics of populations with and without elevated pulse pressure.

	With PP ≥60 mmHg		With PP <60 mmHg		Difference of means	*p* ^c^
	*N* ^a^	Mean (±SD)^b^	*N* ^a^	Mean (±SD)^b^		
Age (years)	2,422	65.00 (12.82)	5,644	55.58 (14.74)	9.42	<0.001
Weight (kg)	2,422	77.91 (15.24)	5,644	76.16 (15.94)	1.75	<0.001
Height (m)	2,422	1.63 (0.09)	5,644	1.64 (0.09)	−0.01	0.360
BMI (kg/m^2^)	2,422	29.25 (5.04)	5,644	28.10 (5.15)	1.15	<0.001
Abdominal girth (cm)	2,394	99.36 (14.86)	5,563	95.21 (14.88)	4.15	<0.001
SBP (mmHg)	2,422	142.93 (13.89)	5,644	123.04 (12.78)	19.89	<0.001
DBP (mmHg)	2,422	75.41 (10.76)	5,644	77.21 (9.81)	−1.80	<0.001
PP (mmHg)	2,422	67.52 (8.92)	5,644	45.84 (8.00)	21.69	<0.001
HR (bpm)	2,422	72.86 (10.94)	5,644	73.50 (10.84)	−0.64	0.013
Fasting blood glucose (mg/dl)^d^	2,422	109.91 (33.36)	5,644	99.04 (25.77)	10.88	<0.001
HbA1c (%)	708	7.14 (1.23)	898	6.95 (1.18)	0.18	0.003
Total cholesterol (mg/dl)^e^	2,422	194.13 (41.35)	5,644	195.60 (55.44)	−1.47	0.127
HDL-C (mg/dl)^e^	2,277	53.73 (15.10)	5,212	55.44 (15.44)	−1.71	<0.001
Non-HDL-C (mg/dl)^e^	2,277	140.74 (40.27)	5,212	141.07 (38.15)	−0.33	0.734
LDL-C (mg/dl)^e^	2,277	116.05 (36.89)	5,212	118.13 (34.81)	−2.08	0.020
Triglycerides (mg/dl)^f^	2,422	131.53 (74.81)	5,644	122.16 (84.19)	9.37	<0.001
Uric acid (mg/dl)	2,099	5.46 (1.48)	4,828	5.16 (1.45)	0.30	<0.001
Creatinine (mg/dl)	2,397	0.90 (0.45)	5,562	0.86 (0.47)	0.04	0.001
eGFR (ml/min/1.73m^2^)	2,397	82.05 (19.51)	5,562	90.38 (19.76)	−8.33	<0.001
ACR (mg/g)	1,759	28.39 (95.86)	3,882	18.61 (65.58)	9.78	<0.001

PP, pulse pressure; BMI, body mass index; SBP, systolic blood pressure; DBP, diastolic blood pressure; HR (bpm), heart rate (beats per minute); HbA1c, glycated haemoglobin A1c; HDL-C, high-density lipoprotein cholesterol; Non-HDL-C, non-high-density lipoprotein cholesterol; LDL-C, low-density lipoprotein cholesterol; eGFR, estimated glomerular filtration rate according to CKD-EPI; ACR, albumin-to-creatinine ratio in urine.

^a^ *N*: sample size.

^b^ SD: standard deviation.

^c^ *p*: *p*-value of the difference of means.

^d^ To convert from mg/dl to mmol/L, multiply by 0.05556.

^e^ To convert from mg/dl to mmol/L, multiply by 0.02586.

^f^ To convert from mg/dl to mmol/L, multiply by 0.01129.

**Figure 2:**
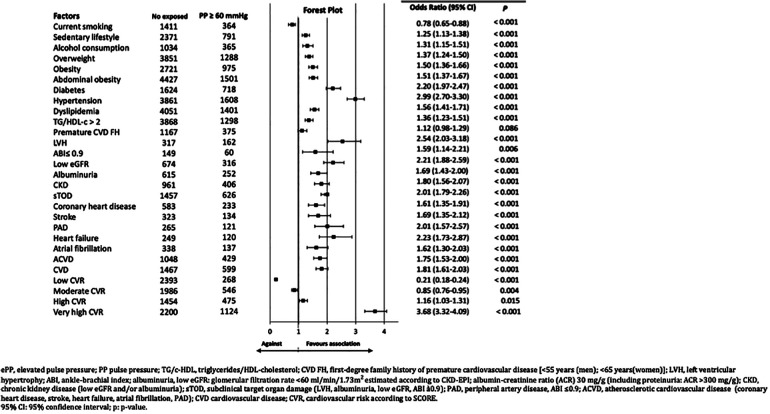
Forest Plot representation of associations between various factors and ePP in the IBERICAN cohort ePP, elevated pulse pressure; PP pulse pressure; TG/c-HDL, triglycerides/HDL-cholesterol; CVD FH, first-degree family history of premature cardiovascular disease [<55 years (men); <65 years(women)]; LVH, left ventricular hypertrophy; ABI, ankle-brachial index; albuminuria, low eGFR: glomerular filtration rate <60 ml/min/1.73 m^2^ estimated according to CKD-EPI; albumin-creatinine ratio (ACR) 30 mg/g (including proteinuria: ACR > 300 mg/g); CKD, chronic kidney disease (low eGFR and/or albuminuria); sTOD, subclinical target organ damage (LVH, albuminuria, low e GER, ABI a0.9); PAD, peripheral artery disease, ABI ≤0.9; ACVD, atherosclerotic cardiovascular disease (coronary heart disease, stroke, heart failure, atrial fibrillation, PAD); CVD cardiovascular disease; CVR, cardiovascular risk according to SCORE. 95% CI: 95% confidence interval; p, p-value.

All the CVRF and the comorbidities assessed were significantly associated with ePP, except the variable first-degree family history of early atherosclerotic CVD (ACVD) and smoking (Table 2). The ePP was significantly (*p* < 0.001) more frequent in patients with HTN (41.65% [95% CI 40.09–43.20]) than in patients without HTN (19.28% [95% CI 18.09–20.48]) (OR: 2.99 [2.70–3.30]), and mainly in patients with SBP/DBP ≥140/90 mmHg (64.66% [95% CI 62.34–66.98]) as compared with those who had SBP/DBP <140/90 mmHg (24.99% [95% CI 23.20–26.78]) (OR: 5.49 [4.78–6.31]). Among the population with HTN, the proportion of patients who had ePP was similar (*p* = 0.678) in men (41.32% [95% CI 39.12–43.52]) and in women (41.98% [95% CI 39.75–44.20]). The other CVRF and comorbidities which showed greater degree of association were LVH, HF, low eGFR (<60 ml/min/1.73m^2^) and DM (Figure 2). In the multivariate analysis, the CVRF and comorbidities which were independently associated with ePP were HTN, LVH, DM, low eGFR, alcohol consumption, abdominal obesity and cardiovascular diseases (Table 3).

**Table 2:** Factors and comorbidities in populations with and without elevated pulse pressure.

	PP ≥60 mmHg		PP <60 mmHg		*p* ^C^
	*N* ^a^	%(95% CI)^b^	*N* ^a^	%(95% CI)^b^	
Current smoking	364	15.03 (13.61–16.45)	1047	18.55 (17.54–19.56)	<0.001
Sedentary lifestyle	791	32.66 (30.79–34.53)	1580	27.99 (26.82–29.11)	<0.001
Alcohol consumption	365	15.11 (13.69–16.54)	669	11.93 (11.08–12.78)	<0.001
Overweight	1288	53.29 (51.28–55.29)	2563	45.50 (44.20–46.80)	<0.001
Obesity	975	40.26 (38.29–42.22)	1746	30.94 (29.73–32.15)	<0.001
Abdominal obesity	1501	62.70 (60.76–64.64)	2926	52.60 (51.29–53.91)	<0.001
Diabetes	718	29.64 (27–83–31–46)	906	16.05 (15.09–17.01)	<0.001
Hypertension	1608	66.56 (64.67–68.44)	2253	39.98 (38.70–41.26)	<0.001
Dyslipidemia	1401	58.04 (56.07–60.01)	2650	47.04 (45.74–48.35)	<0.001
TG/HDL-c > 2	1298	57.00 (54.94–59.02)	2570	49.31 (47.94–50.66)	<0.001
Premature CVD FH	375	16.86 (15.31–18.42)	792	15.27 (14.30–16.25)	0.086
LVH	162	6.69 (5.69–7.68)	155	2.75 (2.32–3.17)	<0.001
ABI ≤0,9	60	2.48 (1.86–3.10)	89	1.58 (1.25–1.90)	0.006
Low eGFR	316	13.18 (11.83–14.54)	358	6.44 (5.79–7.08)	<0.001
Albuminuria	252	10.40 (9.19–11.62)	363	6.43 (5.79–7.07)	<0.001
CKD	406	23.08 (21.11–25.05)	555	14.30 (13.20–15.40)	<0.001
sTOD	626	26.12 (24.36–27.87)	831	14.94 (14.00–15.88)	<0.001
Coronary heart disease	233	9.62 (8.45–10.79)	350	6.20 (5.57–6.83)	<0.001
Stroke	134	5.53 (4.62–6.44)	189	3.35 (2.88–3.82)	<0.001
PAD	121	5.00 (4.13–5.86)	144	2.55 (2.14–2.96)	<0.001
Heart failure	120	4.95 (4.09–5.82)	129	2.29 (1.90–2.68)	<0.001
Atrial fibrillation	137	5.66 (4.74–6.58)	201	3.56 (3.08–4.04)	<0.001
ACVD	429	17.71 (16.19–19.23)	619	10.97 (10.15–11.78)	<0.001
CVD	599	24.73 (23.01–26.45)	868	15.38 (14.44–16.32)	<0.001
Low CVR	268	11.11 (9.85–12.36)	2125	37.81 (36.54–39.08)	<0.001
Moderate CVR	546	22.63 (20.96–24.30)	1440	25.62 (24.48–26.76)	0.004
High CVR	475	19.69 (18.10–21.27)	979	17.42 (16.43–18.41)	0.015
Very high CVR	1124	46.58 (44.59–48.57)	1076	19.15 (18.12–20.17)	<0.001

^a^ N: sample size.

^b^ 95% CI: 95% confidence interval.

^c^ p: p-value.

PP, pulse pressure; TG/c-HDL, triglycerides/HDL-cholesterol; CVD FH, first-degree family history of premature cardiovascular disease (<55 years [men]; <65 years [women]); LVH, left ventricular hypertrophy; ABI, ankle-brachial index; albuminuria, albumin-creatinine ratio (ACR)≥30 mg/g (including proteinuria, ACR >300 mg/g); low eGFR, glomerular filtration rate <60 mL/min/1.73m^2^ estimated according to CKD-EPI; CKD, chronic kidney disease (low eGFR and/or albuminuria); sTOD, subclinical target organ damage (LVH, albuminuria, low eGFR, ABI ≤0.9); PAD, peripheral artery disease, ABI ≤0.9; ACVD, atherosclerotic cardiovascular disease (coronary heart disease, stroke, PAD); CVD (cardiovascular disease), ACVD, heart failure, atrial fibrillation; CVR, cardiovascular risk according to SCORE. *(Consult additional material for reference-checking).*

**Table 3:** Multivariate analysis of risk factors and comorbidities associated with elevated pulse pressure (≥60 mmHg).

ACVD model	*β* ^a^	OR Exp (*β*)^b^	*p* ^c^	CVD model	*β* ^a^	OR Exp (*β*)^b^	*p* ^c^
Hypertension	0.88 (0.06)	2.41 (2.16–2.68)	<0.001	Hypertension	0.87 (0.06)	2.93 (2.14–2.67)	<0.001
LVH	0.48 (0.12)	1.62 (1.27–2.05)	<0.001	LVH	0.46 (0.12)	1.58 (1.25–2.01)	<0.001
Diabetes	0.43 (0.06)	1.53 (1.36–1.73)	<0.001	Diabetes	0.42 (0.06)	1.53 (1.35–1.72)	<0.001
Low eGFR	0.39 (0.09)	1.48 (1.25–1.75)	<0.001	Low eGFR	0.38 (0.09)	1.46 (1.23–1.73)	<0.001
Alcohol consumption	0.22 (0.07)	1.25 (1.08–1.45)	0.002	Alcohol consumption	0.22 (0.07)	1.25 (1.08–1.45)	0.002
ACVD	0.20 (0.07)	1.23 (1.06–1.41)	0.006	CVD	0.20 (0.06)	1.22 (1.07–1.38)	0.002
Central obesity	0.12 (0.05)	1.13 (1.02–1.26)	0.023	Central obesity	0.12 (0.05)	1.13 (1.01–1.25)	0.029

LVH, left ventricular hypertrophy; low eGFR, glomerular filtration rate <60 ml/min/1.73m^2^ estimated according to CKD-EPI; ACVD, atherosclerotic cardiovascular disease (coronary heart disease, stroke, peripheral artery disease); CVD (cardiovascular disease), ACVD, heart failure, atrial fibrillation.

^a^ *β* coefficient (± deviation).

^b^ Odds-ratio Exp (*β*) (95% confidence interval).

^c^ *p*: *p*-value of Wald test with one degree of freedom.

66.27% (95% CI 64.38–68.15) of patients with ePP had a high or very high CVR, as against the patients without ePP, of whom 36.57% (95% CI 35.31–37.83) had a high or very high CVR (OR: 3.41 [95% CI 3.08–3.77]) (Table 2, Figure 2).

## Discussion

This subanalysis of the cohort of the IBERICAN study describes the characteristics of the population according to the presence of ePP, with a prevalence adjusted for age and sex of 23.5%. This prevalence increases with age, is higher in men, and is more frequently associated with HTN, DM, low eGFR, LVH and HF, which increases the CVR of patients with ePP.

After a detailed revision of bibliography, our results represents the first time that are described the prevalence of ePP and its associations with other cardiovascular risk factors, TOD and CVD simultaneously in the same cohort, using a clínica population, recruited in primary care.

The prevalence of ePP observed in our study (23.5%) are similar to observed in other studies as NHANES survey (26.91%) using PP > 55 mmHg in a sample with 5,771 subjects (22) or 12.1% of patients aged <55 and 27.8% of those aged ≥55 in a French study about a sample with 19,083 men (15). The main variable associated with ePP prevalence was the age, with a quasi-perfect linear correlation, variable also associated with the cardiovascular mortality (25), and in older 60 years the ePP has an important predictive value of the cardiovascular risk (26).

The relationship between ePP and other variables as HTN o LVH was described by Vasan et al. that described the association of these TOD with central PP (12). In the same line, other studies analysed the role of ePP in the develop of chronic kidney disease (CKD) (23) or other organ targeting as HF (24). These associations of the ePP can explain that we observed two-thirds of the patients with ePP had higher cardiovascular risk, in the same line observed in the MRFIT study that described the association between PP and cardiovascular mortality in hypertensive patients (27).

These relationships with other cardiovascular risk factor, TOD and CVD describes the ePP as a early risk marker and the importance of and early identification to introduce changes in the treatment of the patients and improve their prognosis. In really, maybe we need more studies, and clinical trials, that confirm that this reduction of PP would reduce the cardiovascular events and mortality.

### Strengths and limitations

This subanalysis of the IBERICAN study has certain limitations derived from its very design and from the interpretation of some of the variables. The study sample has the bias of being a clinical cohort between the age of 18 and 85 seen in Primary Care with a possible accumulation of risk factors and comorbidities as compared with the rest of the population. Thus, the results obtained could be only extrapolated to the clinical population, despite the validity of the associations found. This study does not differentiate whether the ePP was detected during the day or at night, even though the ePP is associated with LVH regardless of the moment of detection whereas the greatest increase of ventricular mass has been associated with ePP during night time. Like SBP and DBP, PP is a continuous variable, so the decision to establish the ePP at an easy-to-remember threshold of 60 mmHg is an arbitrary one, though justified by the available literature (28, 29). The analysis of the variable ACVD (CHD, stroke and PAD) does not differentiate between type-1 (atherothrombotic) and type-2 (non-atherothrombotic) coronary ischemic heart disease, or between ischemic strokes and hemorrhagic strokes. From a strictly clinical point of view, our results can be considered to show the relationship between the set of processes included in the variable ACVD and ePP, because the existence of such association has been previously demonstrated not only with atherothrombotic disease but also with hemorrhagic strokes (30) and with non-obstructive coronary ischemia in stressful situations (31).

Among the strengths of this study are the large sample of the IBERICAN cohort, the adjustment for age and sex of the prevalence rates (which makes it easier to compare the results with other populations), the assessment of the association of ePP with numerous cardiovascular, cardiometabolic and renal variables, and the presentation of relevant results on ePP which did not exist in Spain before.

### Clinical implications

There are no well-designed intervention studies which assess the potential cardiovascular benefits of specific therapeutic strategies for ePP. This may justify the fact that no PP objectives or appropriate treatment has been established. It has been found that the levels of PP reached with antihypertensive treatment form a curved (J-shaped) association for most cardiovascular events, and a linear one when it was associated with myocardial infarction, setting the optimum level of PP at 50 mmHg (32). A strict control of BP lowers the PP levels in varying degrees according to the drug treatment used (Supplementary Figure S3 in the additional material). Emphasis should be placed on individualising HTN treatments, especially in patients with CHD (even in its silent forms), DM or fragile elderly patients, since an excessive reduction of both the SBP and DBP may lead to new cardiovascular events (33–38).

## Conclusions

The observational multicentre IBERICAN study, recruited in primary care in Spain, showed that near a quarter of the patients had ePP, and this prevalence increases with the age of the patients.

The prevalence of ePP showed an independent association with other cardiovascular risk factors, as HTN, diabetes, abdominal obesity and alcohol consumption; other TOD, as LVH and low eGFR; and CVD.

This association with other cardiovascular determinants and the higher cardiovascular risk associated become the ePP in the interesting risk marker to identify in the clinical practice to introduce more intensive treatments to improve the cardiovascular prognosis. However, this affirmation needs to be confirmed in a prospective observational studies and clinical trials.

## Data Availability

The original contributions presented in the study are included in the article/**Supplementary Material**, further inquiries can be directed to the corresponding author/s.

## Group members of the IBERICAN study

The full list of members can be found in the Supplementary material.
