# Supplementary material for: Elevated pulse pressure and cardiovascular risk associated in Spanish population attended in primary care: IBERICAN study
Source: Front Cardiovasc Med. 2023 May 9;10:1090458. doi: 10.3389/fcvm.2023.1090458 (PMC10203900; doi:10.3389/fcvm.2023.1090458)
Supplement: Supplementary file 1 [file Datasheet1.pdf]

Additional Material

● **IBERICAN study**

It is a prospective cohort study of the population seen in Primary Care centres in Spain. It included subjects of either sex, aged 18 to 85, who were selected with consecutive non-probability sampling between April 1, 2014 and October 14, 2018.

- Cinza Sanjurjo S, Llisterri Caro JL, Barquilla García A, Polo García J, Velilla Zancada S, Rodríguez Roca GC, et al., investigators of the IBERICAN study. Description of the sample, design and study methods for the identification of the Spanish population at cardiovascular and renal risk (IBERICAN). Semergen. 2020; 46: 4-15, http://dx.doi.org/10.1016/j.semerg.2019.10.006

- Cinza Sanjurjo S, Prieto Díaz MÁ, Llisterri Caro JL, Barquilla García A, Rodríguez Padial L, Vidal Pérez R, Rodríguez Roca GC, Badimón Maestro JJ, Pallarés Carratalá V. Prevalence of obesity and associated cardiovascular comorbidity in patients included in the IBERICAN study (Identification of the Spanish population at cardiovascular and renal risk). Semergen. 2019; 5:311-322. https://doi.org/10.1016/j.semerg.2018.11.003.

- Cinza-Sanjurjo S, Micó-Pérez R.M, Velilla-Zancada S, Prieto-Díaz M.A Rodríguez-Roca G.C, Barquilla García A, et al. Factors associated with cardiovascular risk and cardiovascular and renal disease in the IBERICAN study (Identification of the Spanish population at cardiovascular and renal risk): final results. Semergen. 2020; 46: 368-78. https://doi.org/10.1016/j.semerg.2020.06.027.

● **Variables considered in this subanalysis**

The following variables were considered: weight (kg); height (m); smoking; sedentary lifestyle; alcohol consumption; body mass index (BMI): weight/height^2 (kg/m^2); overweight: BMI 25.0-29.9 kg/m^2; obesity: BMI ≥30 kg/m^2 [(a)]; abdominal obesity: increased abdominal girth (≥102 cm [men]; ≥88 cm [women])[(b)]; HTN according to the 2018 guidelines of the European Societies of Cardiology and Hypertension (ESC/ESH)[(c)]: systolic blood pressure (SBP) ≥140 mmHg and/or diastolic blood pressure (DBP) ≥90 mmHg, or HTN in medical history; pulse pressure (PP): SBP-DBP; elevated PP (ePP): ≥60 mmHg; heart rate; blood glucose; total cholesterol; low-density lipoprotein cholesterol (LDL-C); high-density lipoprotein cholesterol (HDL-C); Non-HDL cholesterol (Non-HDL-C); triglycerides; TG/HDL-C index; DM according to the American Diabetes Association (ADA)[(d)] or diagnosis of DM in medical history; dyslipidemia: total cholesterol ≥200 mg/dL and/or triglycerides ≥150 mg/dL or record of dyslipidemia, hypercholesterolemia or hypertriglyceridemia in medical history; first-degree family history of

premature cardiovascular event (<55 years [men]; <65 years [women]); atherosclerotic CVD (ACVD); coronary heart disease (CHD) (ischemic heart disease, acute myocardial infarction, acute coronary syndrome, coronary revascularization), stroke (cerebral ischemia, intracranial haemorrhage, transient ischemic attack), peripheral artery disease (PAD) (intermittent claudication, ankle-brachial index (ABI) ≤0.9); cardiovascular disease (CVD): ACVD, heart failure (HF), atrial fibrillation (AF); low glomerular filtration rate (eGFR)[e]: <60 mL/min/1.73 m^2 according to the equation CKD/EPI[f]; albuminuria: albumin-to-creatinine ratio in urine (ACR) ≥30 mg/g (includes proteinuria, ACR ≥300 mg/g); chronic kidney disease (CKD): low eGFR and/or albuminuria[a]; subclinical target organ damage (sTOD): LVH, ABI ≤0.9, low eGFR, albuminuria; low, moderate, high and very high categories of cardiovascular risk (CVR) according to SCORE[g],[h].

References associated with the variables analysed:

[a] Garvey WT, Garber AJ, Mechanick JI, Bray GA, Dagogo-Jack S, Einhorn D, et al; AACE Obesity Scientific Committee. American Association of Clinical Endocrinologists and American College of Endocrinology position statement on the 2014 advanced framework for a new diagnosis of obesity as a chronic disease. Endocr Pract. 2014; 20:977–89, http://dx.doi.org/10.4158/EP14280.PS

[b] Alberti KGMM, Eckel RH, Grundy SM, Zimmet PZ, Cleeman JI, Donato KA, et al. Harmonizing the metabolic syndrome: A joint interim statement of the International Diabetes Federation task force on Epidemiology and Prevention; National Heart, Lung, and Blood Institute; American Heart Association; World Heart Federation; International Atherosclerosis Society; and International Association for the Study of Obesity. Circulation. 2009;120:1640–5. http://dx.doi.org/10.1161/CIRCULATIONAHA.109.192644.

[c] Williams B, Mancia G, Spiering W, Rosei EA, Azizi M, Burnier M, et al., ESC Scientific Document Group. 2018 ESC/ESH guidelines for the management of arterial hypertension: The Task Force for the management of arterial hypertension of the European Society of Cardiology (ESC) and the European Society of Hypertension (ESH). Eur Heart J. 2018; 39:3021–104. http://dx.doi.org/10.1093/eurheartj/ehy339.

[d] American Diabetes Association Professional Practice Committee. 2. Classification and diagnosis of diabetes: standards of medical care in diabetes—2022. Diabetes Care. 2022; 45(Suppl. 1):S17–S38, http://dx.doi.org/10.2337/dc22-S002.

[e] Kidney Disease: Improving Global Outcomes (KDIGO) CKD Work Group. KDIGO 2012 Clinical practice guideline for the evaluation and management of chronic kidney disease. Kidney Int Suppl. 2013; 3:5–14, http://dx.doi.org/10.1038/kisup.2012.77.

[f] Levey AS, Stevens LA, Schmid CH, Zhang YL, Castro AF 3rd, Feldman HI, et al., CKD-EPI (Chronic Kidney Disease Epidemiology Collaboration). A new equation to estimate glomerular filtration rate. Ann Intern Med. 2009; 150:604–12, http://dx.doi.org/10.7326/0003-4819-150-9-200905050-00006.

[g] Piepoli MF, Hoes AW, Agewall S, Albus C, Brotons C, Catapano AL, et al. 2016 European Guidelines on cardiovascular disease prevention in clinical practice. The Sixth Joint Task Force of the European Society of Cardiology and Other Societies on Cardiovascular Disease Prevention in Clinical Practice (constituted by representatives of 10 societies and by invited experts). Developed with the special contribution of the European Association for Cardiovascular Prevention & Rehabilitation (EACPR). Eur Heart J. 2016; 37:2315–81, http://dx.doi.org/10.1093/eurheartj/ehw106.

[h] Cooney MT, Selmer R, Lindman A, Tverdal A, Menotti A, Thomsen T, et al. Cardiovascular disease risk estimation in older persons: SCORE O.P. Eur J Prev Cardiol. 2016;23:1093–103, http://dx.doi.org//10.1177/2047487315588390.

● **Therapeutic strategies associated with the decrease in PP.**

Figure 3. Reduction of PP values achieved with different strategies
Diet / Exercise / Diuretics / ARBs / Ca Antag / ACEi/β-β/ Atorv/ Isosorbide/ Folic/ ASA/ SGLT2i
Diet: Mediterranean diet; Exercise: combined physical exercise (aerobic + resistance); ARBs: angiotensin-II receptor antagonists; Ca Antag: calcium antagonists; ACEi: angiotensin-converting enzyme inhibitors; β-β: Beta blockers; Atorv: atorvastatin; Isosorbide: isosorbide monohydrate; Folic: folic acid; ASA: acetylsalicylic acid; SGLT2i: sodium-glucose cotransporter-2 inhibitors.

Figure 3 has been made from the following references:

- Karpanou, E., Vyssoulis, G., Stefanadis, C. et al. Differential pulse pressure response to various antihypertensive drug families. J Hum Hypertens. 2006;20: 765–771. https://doi.org/10.1038/sj.jhh.1002069.
- Jennings A, Berendsen A, de Groot L, Feskens E, Brzozowska A, Sicinska E, et al. Mediterranean-Style Diet Improves Systolic Blood Pressure and Arterial Stiffness in Older Adults Results of a 1-Year European Multi-Center Trial. Hypertension. 2019; 73:578-586. https://doi.org/10.1161/HYPERTENSIONAHA.118.12259.
- Park W, Jung W-S, Hong K, Kim Y-Y, Kim S-W and Park H-Y. Effects of Moderate Combined Resistance- and Aerobic-Exercise for 12 Weeks on Body Composition, Cardiometabolic Risk Factors, Blood Pressure, Arterial Stiffness, and Physical Functions, among Obese Older Men: A Pilot Study. Int. J. Environ. Res. Public Health 2020; 17: 7233. https://doi.org/10.3390/ijerph17197233.
- Lamarche F, Agharazii M, Nadeau-Fredette A, Madore F and Goupil R. Central and Brachial Blood Pressures, Statins, and Low-Density Lipoprotein Cholesterol. A Mediation Analysis. Hypertension. 2018; 71:415-421. https://doi.org/10.1161/HYPERTENSIONAHA.117.10476.

- Kanaki A, Sarafidis P, Georgianos P, Kanavos K, Tziolas I, Zebekakis P, et al. Effects of Low-Dose Atorvastatin on Arterial Stiffness and Central Aortic Pressure Augmentation in Patients With Hypertension and Hypercholesterolemia. American Journal of Hypertension. 2013; 26: 608-16. https://doi.org/10.1093/ajh/hps098.

- Mitsiou E, Boutari C, Kotsis V, Georgianou E, Doumas M, Karagiannis A, et al. Effect of Low (5 mg) vs. High (20-40 mg) Rosuvastatin Dose on 24h Arterial Stiffness, Central Haemodynamics, and Non-Alcoholic Fatty Liver Disease in Patients with Optimally Controlled Arterial Hypertension. https://doi.org/10.2174/1570161115666170630122833.

- Ichihara A, Hayashi M, Koura Y, Tada Y, Kaneshiro Y and Saruta T. Long-term effects of statins on arterial pressure and stiffness of hypertensives. J Hum Hypertens. 2005; 19: 103–109. https://doi.org/10.1038/sj.jhh.1001786.

- Mangoni A, Ouldred E, Swif C, Jackson E, Draper R, Sherwood R, et al. Vascular and blood pressure effects of folic acid in older patients with cardiovascular disease. Journal of the American Geriatrics Society. 2001; 49: 1003-4. https://doi.org/10.1046/j.1532-5415.2001.49196.x.

- Williams C, Kingwell B, Burke K, McPherson J, and Dart A. Folic acid supplementation for 3 wk reduces pulse pressure and large artery stiffness independent of MTHFR genotype[1–3]. Am J Clin Nutr. 2005; 82:26 – 31. https://doi.org/10.1093/ajcn/82.1.26.

- Stokes G, Barin E and Gilfillan K. Effects of Isosorbide Mononitrate and AII Inhibition on Pulse Wave Reflection in Hypertension. Hypertension. 2003; 41:297-301. https://doi.org/10.1161/01.HYP.0000049622.07021.4F.

- Serenelli M, Böhm M, Inzucchi S, Køber L, Kosiborod M, Martinez F, et al. Effect of dapagliflozin according to baseline systolic blood pressure in the Dapagliflozin and Prevention of Adverse Outcomes in Heart Failure trial (DAPA-HF). European Heart Journal. 2020; 41: 3402–3418. https://doi.org/10.1093/eurheartj/ehaa496.

- Chilton R, Tikkanen I, Cannon C, Crowe S, Woerle H, Broedl U, et al. Effects of empagliflozin on blood pressure and markers of arterial stiffness and vascular resistance in patients with type 2 diabetes. Diabetes, Obesity and Metabolism 17: 1180–1193, 2015.

- Mancia G, Cannon C, Tikkanen I, Zeller C, Ley L, Woerle H, et al. Impact of Empagliflozin on Blood Pressure in Patients With Type 2 Diabetes Mellitus and Hypertension by Background Antihypertensive Medication. Hypertension. 2016; 68:1355-1364. https://doi.org/10.1161/HYPERTENSIONAHA.116.07703.
